# Race, Religiosity, and Neuroticism: Influences on End-of-Life Readiness in Later Life

**DOI:** 10.1093/geroni/igaf122.4180

**Published:** 2025-12-31

**Authors:** Satvika Boyina, Mary Cox, Patrick Hill

**Affiliations:** St. Louis University, St. Louis, Missouri, United States; Washington University in St. Louis, St. Louis, Missouri, United States; Washington University in St. Louis, Saint Louis, Missouri, United States

## Abstract

End of life (EOL) care planning, or preparing for future medical decisions and preferences, is essential for promoting autonomy and quality care in later life. Unfortunately, many individuals delay or avoid this process. This study examined how psychological factors specifically race, racial centrality, religiosity, and neuroticism relate to both EOL planning mindsets and behaviors among older American adults (n = 467; ages 60-88). Data was collected online via Prolific in summer 2025. Participants completed surveys assessing psychological readiness (mindset), planning actions (behavior), personality traits, religious reliance, and racial identity. EOL mindset refers to how psychologically ready or comfortable individuals feel discussing death, while behaviors reflect concrete actions like creating a will or naming a decision-maker. Correlational analyses and t-tests revealed that Black participants reported greater psychological readiness for EOL planning than White participants. Among Black individuals, higher racial centrality was associated with stronger EOL mindsets, suggesting that cultural identity may serve as a motivating or protective factor. Religiosity was also positively associated with the EOL mindset, indicating that spiritual frameworks may enhance emotional comfort with end-of-life discussions. In contrast, neuroticism was linked to both lower EOL readiness and fewer completed planning behaviors, highlighting how emotional vulnerability may act as a barrier to both mindset and action. These findings suggest that sociocultural and psychological factors shape how older adults approach end-of-life planning. For healthcare institutions, these results emphasize the importance of developing culturally responsive and emotionally attuned interventions that foster both readiness in EOL care planning across aging populations.

